# A Treatise on the Causes and Consequences of Habitual Constipation

**Published:** 1840-07

**Authors:** 


### Art. II.

*A Treatise on the Causes and Consequences of Habitual Constipation.* By John Burne, m.d., Fellow of the Royal College of Physicians, Physician to the Westminster Hospital, &c. &c.—*London,* 1840. 8vo, pp. 257.

We view with some timidity any work which brings prominently forward the advantages to be derived from the employment of aperient medicines. There is something so apparently simple in the employment of purgatives; the popular fancy has been so educated into a belief of their necessity in almost every case, and the resort to them is so easy a mode of gaining time to deliberate and (in the common opinion) to prepare the way for the action of other medicines, that we have frequently thought that a book might be advantageously written to caution against, with more advantage than to encourage the use of aperients. From infancy to old age, English bowels are subjected to the influence of unnatural stimuli. The habit acquired by the nurses' repeated dose of syrup of rhubarb and magnesia, is strengthened by the doctor's endeavour to purge away measles, scarlatina, and the majority of children's maladies; the bottle of salts and senna shares with the other instruments of discipline in the management of the youth, every headach from fatigue, every pain or ache from whatever cause being attacked by purgation; while at the more enquiring age, the victim having read in a book that purgatives are "good" for such and such complaints, if he possess not the complaint he imagines it, and forthwith endeavours to expel it by the universal medicine. We believe that it would be difficult to overrate the evils which have been produced in this country by aperient medicines. And we are convinced that there are few subjects in practical medicine which more require on the part of the practitioner a cautious and correct judgment, founded upon a safe scientific principle, than the employment of these. On this account and many others which we might mention, we feel that whilst a book such as that of Dr. Burne may be of advantage in many instances, it cannot be safely read or made the groundwork of principles of practice without the exercise of great caution and judgment. It will be our endeavour, whilst presenting to our readers an abstract of its most valuable contents, to inculcate this caution by occasional observations of our own.

Dr. Burne's plan is, after some remarks upon the physiology of the large intestines, to consider the various consequences of habitual constipation on the general system and on particular parts of it, to discuss its causes and treatment. The last chapter in the volume is devoted to the connexion between constipation and the more prevalent diseases of India.

Imbued with the doctrines of Mr. Abernethy, Dr. Burne commenced his own career of observation of the numerous derangements of the digestive organs.

"I had pursued this enquiry but a short time, when I was struck with the frequency of one particular cause, which could be traced as the only one in operation, from the period of health to the establishment of the disorders the subject of investigation. I found, moreover, that these disorders ceased on the removal of this the presumptive cause, and thence concluded that they stood in the relation of effect to such cause. Next I discovered that this same cause had existed through a long period in diseases apparently only remotely connected with it; and then proceeded to ascertain its relation to these diseases, by tracing back, step by step, the manner of their formation; and the evidence which has resulted authorizes me to attribute the origin and establishment of these diseases to the continued operation of the cause in question; namely, *habitual constipation.*" (p. 2.)

Dr. Burne's experience has been extensive, and the result of it, in many cases illustrative of the theory which he above lays down, is given by the relation of these cases. We doubt not that to every practitioner have occurred some of the examples related by Dr. Burne, whose chief merit consists in the care with which he has recorded a great number of facts, capable, with the exercise of due discretion, of being turned to a valuable practical account.

Dr. Burne speaks of a copious discharge of limpid urine as being frequently diagnostic of a loaded state of the colon. We cannot but consider some cases of melancholy, related by the author as the effect of habitual constipation, as quite destitute of anything like proof of this alleged fact. A great fault of many of the medical writers of this age is the readiness with which small facts are gathered together and fitted, whether right or wrong, to some favorite theory. This is a practice of which we cannot hold our author quite blameless, and against which we must on all occasions strongly protest. There cannot be any possible permanency in theories so constructed, nor any other than an occasional and chance benefit arising from practice founded upon them. It seems to be here forgotten that habitual constipation is abundantly frequent without any melancholy; and the meager relation of the cases referred to might lead us to believe, did we not know the contrary to be the fact, that the author knew nothing in hereditary disposition, in the state of the mind and the affections, in the physical peculiarities of individuals, or in numerous other causes, to which might be ascribed the attendant melancholy.

From the chapter on the effects of habitual constipation on the general health, we extract the following caution as one well deserving attention:

"The period at which young females begin to experience irregularity of the bowels is about the age of puberty. At first, the inconveniences are not such as to attract attention. Slight indisposition happens occasionally, and is attributed to any but the just cause, and unfrequent action of the bowels being thought by ladies at this age rather a desirable habit; but should these pages meet the eye of mothers, I beseech them, as they respect their children's health, to give attention to this subject; and I cannot omit in this place to urge upon all those who charge themselves with the education of the female sex, the same attention as a matter of duty, I would say of humanity; for no one thing is so likely to rouse into action, at this age, pulmonary, hepatic, or any other disease to which the individual may happen to be predisposed, as constipation of the bowels." (p. 34.)

Dr. Burne in this chapter has traced the effects of constipation in its earlier and later stage. In much that he has said we cordially agree, though we think that, as in previous instances, he has not always duly discriminated between the *post* and the *propter*. The disease termed "sick headach" is regarded by Dr. Burne as most commonly caused by habitual constipation. His cases are worthy of perusal. This dependence of the headach and the other train of symptoms upon the constipation is regarded as being proved by the fact that recovery of health is in some instances procured "through means adapted only to the regulation of the bowels." But to ourselves, we must repeat it, the proof is still wanting. An attack of "sick headach" appears frequently to be an effort, and generally a successful one, to relieve the system of some noxious matter. If by the administration of purgatives which produce dejections "two or three times every morning," the bowels having been previously constipated, the disease is cured, this does not prove that mere constipation was the cause. We should be quite as much disposed to infer in these cases, that by the "two or three dejections every morning" the system was cleared of noxious matter just as it had previously been relieved of it by the spontaneous actions which so commonly accompany, or form a part of the affection termed "sick headach;" the purgatives being not alone the means of remedying constipation, but of cleansing the fluids of the system generally. And we the more urge our suggestion on this point, because the treatment must vary materially according to one or other view of the case. Dr. Burne's theory would lead him mainly to a reliance on aperient medicines as remedies for constipation; the theory just stated would lead us to recommend such a change in the habits of life, and especially of diet, as would prevent the formation of such matters as require a violent effort of the system for their discharge. We by no means wish to leave out of view the influence of constipation; on the contrary, we believe it to be a frequent remote cause of this affection; but we think that Dr. Burne has given it a preeminence which it does not deserve. The author takes no notice of the powerful influence of mental causes, or causes operating directly on the nervous centres, which we regard as of equal frequency, and as of much greater importance than constipation.

Speaking of *indigestion, gastrodynia, pyrosis, and sub-acute inflammation and organic diseases of the stomach*, Dr. Burne remarks, "I do not advance the opinion that habitual constipation is the exclusive cause of the above affections, but that it is the most frequent; a point I urge the more, that, in the treatment of these affections, remedies may be directed to the relief and removal of the constipation as an essential and chief means of relieving and removing the disorders of the stomach."

There are many instructive cases of the above-mentioned affections related by Dr. Burne, to which, with the same cautions which we have already given, we may refer our younger readers.

"The treatment [of gastrodynia and pyrosis] is conducted on the principle of restoring the natural function of the bowels, of appeasing the irritability of the stomach, of subduing the subacute inflammatory action, and of removing, as far as possible, its consequence on the tissues of the stomach. The means adopted have been *calomel, opium, the local abstraction of blood, counter-irritation, and appropriate aperients*...........The calomel has been given in the dose of *half a grain*, and the opium of *a quarter of a grain* twice a day; namely, after breakfast and after dinner. At these periods the opium reconciles the stomach to the presence of food, and the calomel acts gradually as a mercurial. The dose of mercury is small in order that it might not salivate too soon; time being required to remove diseases of long standing. The effect of this combination is most happy; and success attends the treatment of almost every case which admits of recovery. The medicines are persevered in for many weeks unless the gums become affected, when the calomel is omitted, but the opium is continued..... Where the patients have not yet suffered much emaciation, the *abstraction of blood* by cupping to a moderate amount, as *four or six ounces*, from the region of the stomach, very much aids the cure; as does also the repeated application of *blisters*.........Aperients must be selected according as any occasional peculiarity exists. I prefer *senna* or *magnesia* with the *compound decoction of aloes*, sometimes with the *sulphate of magnesia*, or *rhubarb* with *magnesia* or *sulphate of potass*.........Whatever may be the aperient or purgative required, the bowels must be kept open.......The *diet* should consist strictly of food of the plainest description, and known to be of easy digestion. Broths seldom agree so well as solid food. Vegetables should be well done, and if perfectly tender may be eaten in moderation. Everything that is rich should be avoided. Cream, butter, and sugar should be used sparingly. Strong wines and spirits will frequently afford a momentary relief, but they are nevertheless injurious, and should be abstained from altogether. Perry, in the quantity of a wine-glassful twice a day, where the weakness is great, has proved agreeable and beneficial." (pp. 85-7.)

We have given the above treatment with some minuteness, because we think it, as medical treatment, not to be despised; although we think also that mercurialization is recommended somewhat too generally and liberally. We are of opinion, moreover, that a mode of treatment more strictly dietetic will often produce as speedy a cure and generally a more lasting one. We are ourselves in the habit of modifying this regiminal treatment very greatly according to the varying circumstances of the case; but an experienced and ingenious friend of ours adopts the following method in almost every case, and, as he states, with singular and almost uniform success: Let the breakfast, he says, be of stale bread and a rasher of bacon, with well-made cocoa, or milk and water in small quantity; the dinner of one or two tender mutton chops, with stale bread and a little weak brandy and water; let tea be avoided as if it were poison; and for supper allow the patient to take an egg or two softly boiled, with stale bread and some weak brandy and water or a glass of good sherry. Begin, if you please, by clearing out the bowels with a good dose of rhubarb; and if the stools be fetid and of bad colour repeat the dose. Let as much exercise be taken as possible. Enjoin, with early rising, the daily employment of the flesh-brush and sponging of the body with cold water, or brine, or vinegar and water; and offend the stomach by as little physic as possible.

We think that there is much valuable matter in the chapter on "The Consequences of Habitual Constipation on the Sexual Organs in the Female, through the sympathy of contiguity." The diseases referred to this cause are "*hyperæsthesia and hypertrophy of the womb, dysmenorrhœa, menorrhagia, amenorrhœa, leucorrhœa, abortion, miscarriage, supposed pregnancy.*" It is observed "that habitual constipation ought to be recognized as one very frequent, perhaps the most frequent cause of affections of the sexual organs in females; and even where it is not absolutely the cause, that it is always a source of aggravation." The fact of the rectum, when its normal function, as explained by Professor O'Beirne, is interrupted, becoming the receptacle for fecal matter which is retained therein, and which becomes a cause of irritation by contiguous sympathy, is very properly insisted on by Dr. Burne.

"This irritation is propagated to the other viscera of the pelvis, and an abnormal quantity of blood is determined to them also. The effect of this determination or hyperæmia is to heighten the sensibility of parts, and to cause their functions to be performed with sensibility and pain. Hence *hyperæsthesia* or *tenderness* of the womb; hence *the sense of heat about the rectum, pudenda, and bladder;* hence *dysuria* and *dysmenorrhœa,* and, I may add, *painful sexual intercourse and difficult parturition.*" (p. 92.)

In most of the instances of *prurigo pudendi muliebris et podicis* which have fallen under Dr. Burne's notice, long-existing habitual constipation has preceded them. *Hypertrophy, menorrhagia,* and *leucorrhœa* are traced to the same cause, and also the irritable uterus described by Dr. Gooch. In many of these instances, where Dr. Burne thinks that habitual constipation has been the cause of the disease, he does not maintain that attention to the bowels alone is sufficient for their cure, although this must constitute the most important part in the treatment. Ergot of rye he regards as of great use in menorrhagia.

"I have heard persons express doubts of its efficacy; but so many cases under my own care have been benefited or cured by it, that I cannot but regard it as a most valuable addition to the materia medica. .........Cases in which an exhausting draining hemorrhage has persisted for five or six weeks after abortion have yielded at once to the influence of the ergot." (p. 102.)

It is very important that the ergot should not be at all in a state of decay.

Our own experience bears out the author's in the benefit derived from belladonna in dysmenorrhœa.

"In the treatment of painful menstruation I have found the belladonna most valuable. I have usually prescribed it in the dose of a *quarter of a grain of the extract,* made into a small pill, which may be taken twice a day, where patients suffer, but not very severely, for two or three days about the menstrual period. In the more urgent cases I advise it to be repeated in one hour; then again after an interval of two; then of three hours; till two, three, or four doses have been taken, according to circumstances: and I have seldom been disappointed in the result. It may produce giddiness and dimness of sight, but they soon pass away. Its unpleasant effects are less then opium, and its efficacy decided." (p. 103.)

Dr. Burne alludes to the *pain in the left side, about the hypochondrium,* which is an ordinary concomitant of leucorrhœa, on the connexion of which with the female sexual organs, he remarks, "so constant is this association, that when a patient suffers from pain in the left side, about the margin of the false ribs, it is almost certain that she is affected either with leucorrhœa, or irritable uterus, or prolapsus, or is pregnant." The author distinguishes gonorrhœa from leucorrhœa by the condition of the inguinal glands, for "in gonorrhœa they are almost invariably involved more or less," whereas they have never been observed by Dr. Burne to be affected in leucorrhœa.

*The liquor aluminis compositus* diluted, as a *wash* not as an *injection*, and applied to the pudenda, the labia being separated, "restrains and cures leucorrhœa. The readiness and almost certainty with which leucorrhœa yields to this outward application, has in part led me to infer the source of leucorrhœa to be from the glands of the os externum." We will not stop to enquire how far the fact of the almost certain cure of leucorrhœa by the application of an astringent wash to the os externum supports the theory of the dependence of that disease on habitual constipation—et sic de cæteris.

From the next chapter we shall but select the following case, as food for very serious reflection to a certain class of surgeons who are extremely fond of cases of stricture of the rectum, and of curing them by means of bougies. The case occurred in the practice of Dr. Roots.

"The doctor was called in great haste, one morning, to a gentleman, whom he found in a most alarming state from depression of the vital powers, and indescribable pain in the abdomen. The abdomen was tense and tender, the pulse was scarcely perceptible; the skin was clammy; and the countenance anxious and haggard: all indicating the utmost danger. The surgeon in attendance reported that the symptoms arose from spasm, and informed the doctor that the patient had *two* strictures of the rectum, which, for some time past, he had been treating with the bougie; that one had yielded; that the patient having been out of town returned last evening; and that he, the surgeon, endeavoured, as usual, to pass the bougie, but could not succeed, on account of a spasm of the gut; that he persevered, when suddenly the patient complained of violent pain, and soon afterwards became alarmingly ill; that he had been with him all night, and had administered injections without avail; nothing, except a little blood and mucus having been evacuated. Dr. Roots gave an unequivocal opinion that the symptoms arose from rupture of the bowel, and that a fatal issue was at hand. The gentleman died in the course of the day. The body was examined; and a lacerated perforation of the rectum discovered. Peritonitis had ensued, and castor oil, administered in the enemas, was seen in the cavity of the abdomen. There was no stricture." (p. 150.)

Dr. Burne thinks that erythema nodosum, psoriasis, impetigo, and acne are the cutaneous eruptions more particularly connected with habitual constipation.

Among the "causes of habitual constipation," Dr. Burne mentions especially inattention to the calls of nature, the inconveniences arising from the want of proper water-closets and privies, the interference with the natural bodily necessities by some of the results of what is absurdly called civilization—sedentary habits and literary pursuits. Certain pathological and mechanical causes are likewise alluded to, together with some of a purely mechanical operation. It is well to bear in mind that magnesia, chalk, cubebs, white mustard seed, pills, the sesquioxyde of iron, adulterated bread, various concretions, fruit stones, &c. may be the mechanical obstacles to the passage of fæces through the bowels.

We shall terminate our notice of Dr. Burne's work by such extracts from his remarks on the treatment of habitual constipation as may be useful to our younger readers. The author very properly insists upon the necessity of ascertaining the peculiar disposition of any individual before determining on the propriety of any interference with his bowels. It is known to everybody that cases are occasionally met with where the bowels are only evacuated every second, third, or fourth day, or even much more seldom, the health of these individuals, however, remaining sound; and on the other hand there are abundant instances in which a relaxed state of the alimentary canal appears to be the essential condition of health. We extract the following as a very useful practical caution on the subject of idyosyncrasies in general:

"To these peculiarities, when stated, we are too little disposed to listen; we are apt to regard them as caprices and fancies rather than as true idyosyncrasies, until some untoward circumstances admonish us that they cannot be slighted or disregarded without hazard to the well-being of our patients and to the reputation of ourselves." (p. 195.)

Under the head of *regimen*, as the first circumstance in the management of constipated bowels, early rising is recommended, and with a wise definition of what this means.

"By early rising, I would understand rather the avoiding a second sleep in the morning than the getting up at any specified hour. ........... Early rising must be construed relatively......... A person awakes refreshed, light, cheerful; but if, instead of at once getting up, he dozes off to sleep again, he afterwards rises with unwillingness, and finds his head heavy, his spirits dull, and his bowels indisposed to act...........Next to early rising and not less important is the *habit of frequenting the closet regularly at a certain period of the day, and of strictly obeying the calls of nature*." (p. 196.)

The subject of diet is one of great importance in relation to constipation; but Dr. Burne has said nothing at all novel on the subject. The author does not think that attention proportioned to its importance has been given to the subject of aperient medicines in habitual constipation. He well remarks that the proper object is attained "not by purging the bowels, but by securing their full and free action at regular periods by medicines which not only act but which dispose the bowels to act of themselves." This most invaluable rule cannot be too strongly impressed upon the minds of every one who contemplates the employment of aperient medicines of any kind.

"I have generally found it better," he adds, "at the commencement, to administer aperients in sufficient dose every other day, taking the chance of the bowels relieving themselves on the alternate day, until they have been brought into a more tractable state, and the influence of medicines upon the individual ascertained; after which the aperient can be so regulated in dose as to be administered daily with advantage. Perseverance on the part of the patient is absolutely necessary...........In proportion as the state of the bowels improves and becomes more tractable, so let the dose of the aperient be diminished, till at length little and eventually none shall be required. I have known persons obliged to commence with an ounce of infusion of senna who have been gradually able to reduce it to a teaspoonful. So with castor oil, dinner pills, and the like." (p. 206.)

Those who suffer from piles are properly recommended so to take aperient medicines as "to have the bowels relieved in the evening, because they soon afterwards go to bed, and their sufferings, which have been aggravated by the action of the bowels, are relieved by the horizontal position: whereas, if the bowels act in the morning, the irritation arising therefrom is kept up during the day by exercise and the erect position." On this account it is recommended that sulphur, the best aperient in such cases, combined with a little magnesia, should be taken about noon. In the chapter "on the Action and Value of Aperient Medicines, administered singly or in combination," there is not much that is new, although there is that which it is useful frequently to repeat. *Jalap* is an aperient thought well of by Dr. Burne. Combined with rhubarb in the form of pills, or mixed with the confection of senna, he has found it very efficacious.

"As an occasional purgative, where the bowels are foul, as in persons on the eve of having an attack of gout, the following jalap draught, taken fasting in the morning, clears the alimentary canal most beneficially and without distress: ℞ Pulv. Jalapæ, ʒss; Vini Colchici, Tinct. Hyoscyami, Tinct. Lavand. C. singulor. ʒss, Aquæ Distillatæ, ℥j." (p. 212.)

*Aloes* is said to be "very certain in its operation, but objectionable as a general remedy in habitual constipation, because it leaves the bowels disposed to be confined, so that no ground is gained beyond the immediate relief; and because when taken continually it rather loses its effect, and requires the dose to be augmented."

The following form of dinner pill is recommended by Dr. Burne: ℞ Aloes, ʒj., Pulv. Rhæi, ℈ij., Pulv. Ipecac. gr. v., Mellis, gr. xij., Spirit. Tenuioris, q. s. M. et in pil xx, vel xxx, vel xl. divide, ex quibus unam, duas, vel tres paulo ante prandium quotidie sumat." The compound decoction of aloes is less irritating in its operation than the aloes given in substance. The author is partial to a compound of this medicine with Epsom salts, which he speaks of as one of the most useful in habitual constipation. Assuredly it is one of the most nasty. However, as there is a variety of tastes among mankind, those who are so disposed may try this: "℞ Magnesiæ Carbonatis, ʒjss, Magnesiæ Sulphatis, ʒvj., Decocti Aloes, C. ℥ij., Aq. Distillatæ, ℥vj. M. Cochlearia ij. vel iij. majora semel bisve quotidie."

*Calomel* is objected to altogether by Dr. Burne as an aperient, in the treatment of habitual constipation; not that he excludes it from use in certain cases of complication with torpid liver, &c.

"Castor oil," he says, "is on the whole one of the most innocuous and certain aperients..........It acts quickly, does not produce a subsequent costiveness, and the longer it is given the less the dose required; a great desideratum."

*Senna* is a purgative approved of by Dr. Burne, especially on account of its repeated doses admitting of diminution without a lessening of its aperient operation.

Dr. Burne thinks that the *bougie* may be had recourse to more frequently than is customary in the treatment of habitual constipation; that as action of the bowels in infants is frequently much promoted by the introduction of a small candle, a piece of soap, &c. the bougie is equally useful to adults. The author objects, and we think with justice, to the employment of clysters as an habitual remedy.

"In the first place they do not continue to relieve the bowels fully and freely for any length of time; in the next place they do not dispose the bowels to resume their natural action, but on the contrary, render them more confined; in the third place they wash off the mucus from the intestine, which is followed by a degree of irritation and an unpleasant sense of heat, very similar to that which occurs after washing the hands in water simply; in the fourth place the fæces become more scybalous and hard under their use; and lastly, the individual does not feel the comfort or conviction of having had his bowels fully relieved, on which account he is often induced to resort to a second lavement on the same day. Lavements fail in completely obviating or curing habitual constipation." (p. 229.)

As an occasional resource, however, our author does not reject them. He recommends the injection of the blandest fluids, such as *barley-water, thin gruel, linseed tea, or milk and water*, but simply warm water he regards as acting injuriously upon the mucous membrane of the rectum, and he prefers the use of water of a temperature of 60° F.

In the treatment of obstructed bowels from fæces or foreign substances accumulated in the cæcum or colon, he wisely cautions against the too frequent employment of purgatives.

"If the cæcum or colon is the seat of the obstruction, a tumour may generally be distinguished in the right ilio-inguinal region, or in the region of that part of the colon where the obstruction is seated, which is most generally the sigmoid flexure. The general plan of treatment should be to abstract blood, more or less, from a vein, if the symptoms call for it, and also locally; secondly, to give one or two strong doses of purgative medicine, as colocynth and calomel followed by senna and sulphate of soda: but if these fail, they should be discontinued, and the effervescing saline aperients resorted to and persevered in; opium or the salts of morphia being at the same time administered to remove spasm and assuage pain. The first efforts not having been successful, time should be allowed; and fomentation and baths and clysters be employed as far as the patient's strength will admit. Treated on this plan, patients will survive and do well after many days (*ten* I have known) of actual obstruction with vomiting and hiccup: but if violent measures are persisted in, they will too often sink under the treatment rather than the disease." (p. 233.)

When the obstruction is in the sigmoid flexure of the colon, the injection of fluids through a long gum elastic tube, as recommended by O'Beirne, is the most effectual mode of overcoming it. When in the rectum, mechanical means should be employed, and "the sign which in cases of obstruction should excite suspicion that the cause is seated in the rectum and lead to an examination of that gut, is tenesmus." This fact, although familiar to all practitioners of experience, is often overlooked by young practitioners.

In speaking of the sympathies existing between the pelvic viscera, Dr. Burne quotes an observation of Sir Charles Clarke, that "in proportion as the practitioner is engaged in treating the complaints of one or the other of these parts, he will be led to attribute the symptoms to that organ to which his attention has been chiefly directed." With this caution borne in the reader's mind, and leading him to a constant scrutiny of Dr. Burne's cases and opinions, both in forming an opinion of them and in making them the groundwork of any principles of practice, we think that the work, the notice of which we have now concluded, may be read with considerable advantage. To the man of experience and reflection there is not much that is novel in it; the tendency of the mode and character of the medical education of this century having been to excite the same train of thought and lead to the same kind of practice in the majority of instances; still we think the book a very useful one, as a remembrancer to the old and guide to the young practitioner; and we can

LIBRARY OF THE

Orleans Parish Medical Society conscientiously recommend it to the best attention of our readers. The general practice inculcated in it, when conducted with great care, is undoubtedly most beneficial; it is its tendency chiefly to lead to excess, against which we would warn our readers. Among the guides to the proper employment of aperient medicines, we do not think that Dr. Burne has given sufficient attention to the colour and general character of the discharges. Making all allowance for peculiarities produced by the nature of the ingesta in the excrementitious matter, there are certain variations of the greatest importance in determining on the propriety of continuing the employment of aperient medicines. In children especially are these noticeable; but there are few chronic diseases even of adults, certainly none connected with a disordered state of the alvine excretion, which can be successfully or even safely treated without a careful and systematic inspection of the stools.

---

